# Comparative Study on Welding Characteristics of Laser-CMT and Plasma-CMT Hybrid Welded AA6082-T6 Aluminum Alloy Butt Joints

**DOI:** 10.3390/ma12203300

**Published:** 2019-10-11

**Authors:** Zhibin Xin, Zhibin Yang, Han Zhao, Yuxin Chen

**Affiliations:** 1 School of Materials Science and Engineering, Dalian Jiaotong University, Dalian 116028, China; 2 Engineering Department of CRRC Sifang Co., Ltd, Qingdao, Shandong 266111, China

**Keywords:** laser-CMT hybrid welding, plasma-CMT hybrid welding, aluminum alloy, microstructure, mechanical property

## Abstract

Laser-CMT (Cold Metal Transfer) and plasma-CMT hybrid welding are two promising alternative joining technologies for traditional Metal-Inert-Gas (MIG) welding of the aluminum alloy joints in the high speed trains manufacturing industry. In this work, a comparative study on the weld formation, microstructure, micro-hardness, and mechanical properties of the butt joints in the two welding methods was conducted. The results indicate that the overall quality of the laser-CMT and plasma-CMT welds were good, especially of the laser-CMT hybrid weld, and the laser-CMT hybrid welding process needed a lower heat input. The width of the partially melted zone of the laser-CMT hybrid weld was narrower than that in the plasma-CMT hybrid weld. Micro-hardness test results show that two distinct softening regions were identified in the heat affected zone, and the micro-hardness values of each zone in the laser-CMT hybrid weld were lower than that in the plasma-CMT hybrid weld. The tensile strength of the laser-CMT hybrid welded joints was higher than that of the plasma-CMT hybrid welded joints, which could reach up to 79.4% and 73.7% of the base materials, respectively. All the fractures occurred in the softening region and exhibited a ductile shear fracture with a shear angle of approximately 45°. The fractographs manifested that the laser-CMT and plasma-CMT hybrid welded joints presented ductile fracture and ductile-brittle fracture features, respectively.

## 1. Introduction

Aluminum alloys have been widely applied in the high speed trains manufacturing industry for many years due to their high strength-to-density ratio and good machinability as well as their excellent weldability [1,2]. Up to now, the traditional Metal-Inert Gas (MIG) Welding remains to be the dominant joining technology adopted for joining the aluminum alloy structures in this field, but its obvious drawbacks are large welding deformation, serious joint softening, and low production efficiency [3,4]. Aluminum alloys, because of their strong thermal conductivity and large thermal expansion coefficient, the divergent heat source, and low energy density during their MIG welding process, are the main reasons for the disadvantages mentioned above have been generally recognized [5,6,7,8].

Considering the heat source characteristic, laser-MIG and plasma-MIG hybrid welding are two promising alternative joining technologies for the traditional MIG welding, because of their high energy density can easily reduce welding heat input and increase welding speed; meanwhile, the welding deformation and the joint softening tendency are also obviously decreased [9,10]. Previous studies of the laser-MIG and plasma-MIG hybrid welding of aluminum mainly focus on the optimization of welding parameters, defects formation mechanism, microstructure, and mechanical properties, the results indicated that the laser-MIG and plasma-MIG hybrid welding are high quality and efficient welding technologies [11,12,13,14,15,16]. 

Compared to the MIG welding, Cold Metal Transfer (CMT) welding is a new welding technology with lower welding heat input [17]. Therefore, laser-CMT and plasma-CMT hybrid welding should be more suitable to reduce welding deformation and joint softening. However, only a small amount of studies have drawn attention to laser-CMT hybrid welding [18], much less for plasma-CMT hybrid welding, especially lack of comparative study of those two new hybrid welding technologies.

In the present work, laser-CMT and plasma-CMT hybrid welding were performed upon 6 mm thickness AA6082-T6 aluminum alloy butt joints using the optimized welding parameters, respectively. The main goal of this research is to comparatively investigate the weld formation, microstructure, micro-hardness, and mechanical properties of the butt joints in the two welding methods, in order to select reasonable welding technology for high speed trains manufacturing industry.

## 2. Materials and Experimental details

### 2.1. Materials

**Figure 1:**
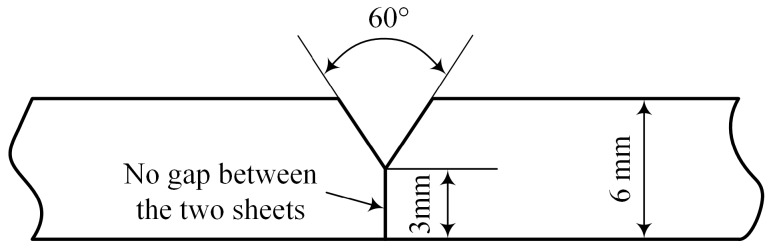
Groove geometry of the welding specimens.

The base material used in the present study was AA6082-T6 aluminum alloy with a thickness of 6 mm, which is widely used in the high speed trains. The plates were machined to the dimensions of 200 mm × 150 mm × 6 mm, geometry design of the grooves is shown in Figure 1. It is noteworthy that there was no gap between these two sheets during the hybrid welding processes. The filler wire was ER5356 with a diameter of 1.2 mm. The chemical composition of the base material and filler wire are given in Table 1. 

Before welding, in order to remove the oxides and decrease the porosity of the weld, the pre-treatment of the base materials was conducted using mechanically cleaning followed by scrubbing with acetone. The storage duration of the pre-treated materials must be less than 24 h. 

### 2.2. Welding Method and Apparatus

**Figure 2:**
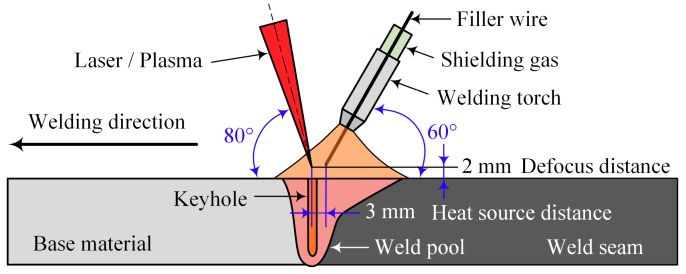
Schematic diagram for experimental setup (not to scale).

In this study, two kinds of hybrid welding methods, including laser-CMT and plasma-CMT hybrid welding were performed to join the AA 6082-T6 aluminum alloy sheets. The laser-CMT hybrid welding experiments were carried out by an IPG YLS-6000 fiber laser (Burbach, Germany) in combination with a FRONIUS TPS 500i CMT welding machine (Wels, Austria). The fiber laser with an emission wavelength of 1.06 μm, the laser beam passed through a focusing lens with a focal length of 300 mm and was finally focused into a spot with a diameter of 0.2 mm. The plasma-CMT hybrid welding experiments were carried out by a super-MIG welding system in combination with a FRONIUS TransPuls Synergic 5000 CMT welding machine(Wels, Austria). The schematic diagram for the two experimental setups is illustrated in Figure 2. During the hybrid welding process, the angles of the laser (plasma) beam and CMT torch to the workpiece were 80° and 60°, respectively. The defocus distance was ±2 mm, the heat source distance was 3 mm, and 99.999% pure argon with a flow rate of 20 l/min was used as shielding gas. 

In order to facilitate comparison, the two hybrid welding experiments were performed using their optimized welding parameters, respectively. During the hybrid welding, the weld penetration need reassurance, and considering weld appearance and the porosity of the joint, the optimized welding parameters are shown in Table 2. In addition, for better comparative the effects of laser and plasma during the hybrid welding process, the CMT current and CMT voltage adopted was basically the same.

### 2.3. Testing Examination 

**Figure 3:**
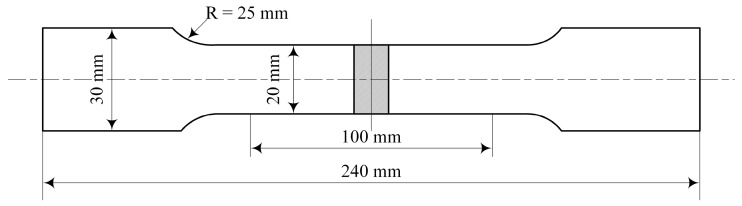
The geometric dimensions of the tensile specimen.

The metallurgical samples were prepared by standard procedures and etched using Keller’s reagent (1 ml HF, 1.5 ml HCl, 2.5 ml HNO_3_, and 95 ml H_2_O) for about 10 s at room temperature, and then, the metallographic analysis was performed by an optical microscopy (OLYMPUS, BX51M, Tokyo, Japan). The micro-hardness was measured using a Vicker micro-hardness tester (FUTURE-TECH, FM-700, Kawasaki, Japan) with a loading force of 100 g for 10 s. The tensile specimens were prepared according to the standard of ISO 4136: 2001 [19], and the geometric dimensions of the tensile specimen is shown in Figure 3. The tensile testing was carried out with a stretching rate of 2 mm/min at room temperature using a universal tensile machine (WDW-300E, Jinan, China), and the tensile strength was the average of three specimens. The tensile fracture surface morphology was observed by a scanning electron microscope (ZEISS, SUPRA 55, Jena, Germany).

## 3. Results and Discussion

### 3.1. Macro and Microstructure

**Figure 4:**
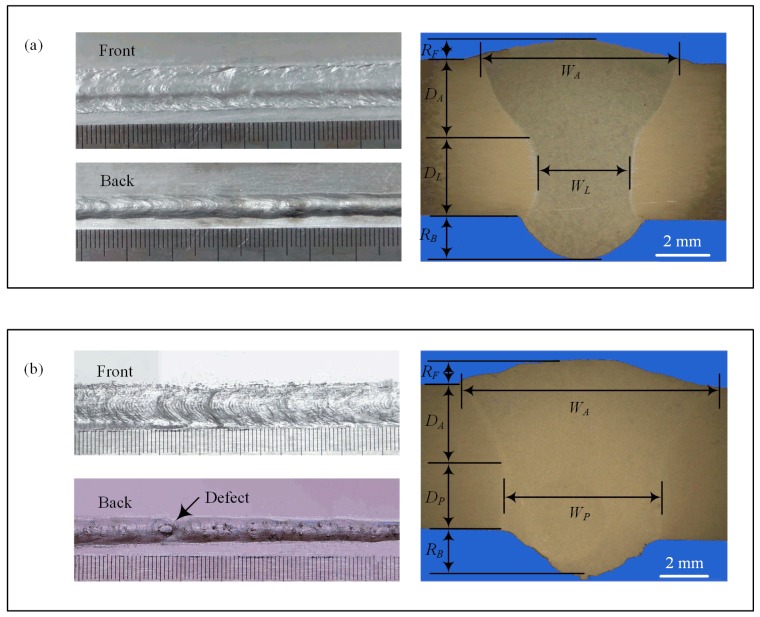
Cross-sections and weld appearances obtained with their optimized welding parameters. (**a**) laser-CMT hybrid weld; (**b**) plasma-CMT hybrid weld.

Figure 4 shows the weld appearances and cross-sections of the optimized laser-CMT and plasma-CMT weld with their optimized welding parameters as given in Table 2. In general, the overall quality of the welds was good. For the weld surface appearances, as shown in Figure 4a,b, the front surfaces of the both hybrid welds were uniform and hardly any spatters appeared, which because the droplet transfer in CMT arc mode resulted in the impact of droplets on the weld pool was weakened and the melt flow was stabilized [20]. However, the plasma-CMT hybrid weld border was not as clean as that of laser-CMT hybrid weld; moreover, the back surface formation quality of the plasma-CMT weld (even appeared defect) was also not as high as that of laser-CMT hybrid weld. Therefore, by comparison, the weld appearance of the laser-CMT hybrid welding was better than that of the plasma-CMT hybrid welding. For the weld cross-section appearances, compared with the plasma-CMT hybrid weld, the laser-CMT hybrid weld appeared “funnel shaped” and could be more obviously divided into two zones: laser zone and arc zone [21]. 

For the sake of convenience, the characteristic dimensions and zones of the weld cross-section were defined as follows: arc zone depth (*D*_A_), laser zone depth (*D*_L_), plasma zone depth (*D*_P_), arc zone width (*W*_A_), laser zone width (*W*_L_), plasma zone width (*W*_P_), weld front surface reinforcement (*R*_F_), and weld back surface reinforcement (*R*_B_); the geometric dimensions of each zone were listed in Table 3. By comparison, to obtain the fully penetrated weld, the heat input of laser-CMT hybrid welding process (3.34 kJ/cm) was lower than that of plasma-CMT welding process (5.92 kJ/cm); this indicated that the laser-CMT hybrid welding was lower cost and energy efficiency. At the same time, the area of laser-CMT hybrid weld was smaller than of plasma-CMT hybrid weld.

**Figure 5:**
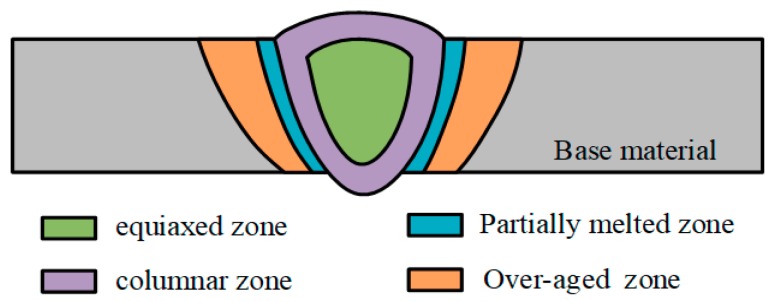
Diagrammatic drawing of the five distinct zones observed in the weld cross-sections of the hybrid welded joint (not to scale).

From the weld center to the base material, micrographs of the weld cross-sections indicated that both of the laser-CMT and plasma-CMT hybrid welded joints consisted of five distinct zones as follows: equiaxed zone (EZ), columnar zone (CZ), partially melted zone (PMZ), over-aged zone (OZ), and base material (BM), as shown in Figure 5. 

**Figure 6:**
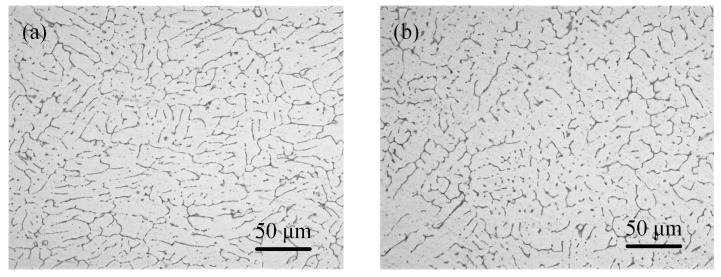
Microstructure at the center of the weld. (**a**) Laser-CMT hybrid weld; (**b**) plasma-CMT hybrid weld.

The equiaxed dendrites were identified both in the laser-CMT and plasma-CMT hybrid weld center, as shown in Figure 6. By contrast Figure 6a,b, the sizes of equiaxed dendrites in laser-CMT hybrid weld was smaller. It is well known that the size of microstructure usually depends on heat input [22]; therefore, the reason of this difference is that the heat input of laser-CMT hybrid welding was lower. Meanwhile, it is important to note that the equiaxed grains formed in the laser-CMT hybrid weld was more fine and uniform than which formed in the plasma-CMT hybrid weld.

**Figure 7:**
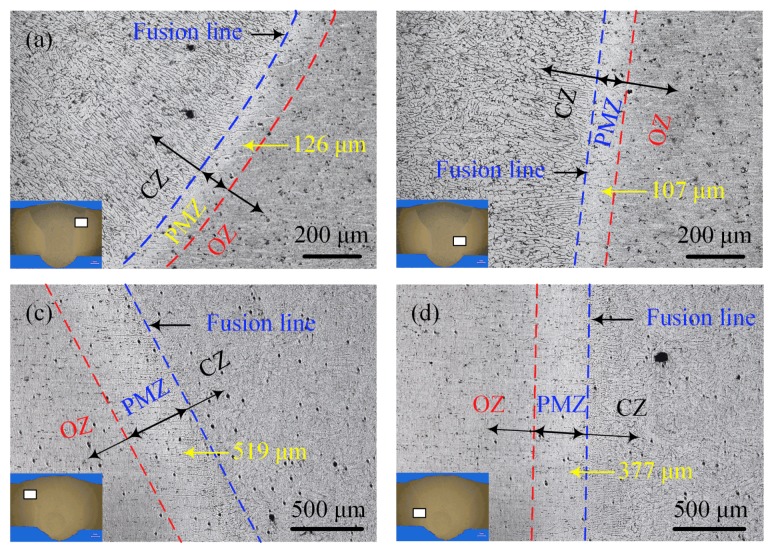
Microstructure near the fusion line. (**a**) arc zone of laser-CMT hybrid welded joint; (**b**) laser zone of laser-CMT hybrid welded joint; (**c**) arc zone of plasma-CMT hybrid welded joint; (**d**) plasma zone of plasma-CMT hybrid welded joint.

Near the fusion line, the microstructure of the laser-CMT and plasma-CMT hybrid welded joints had something in common; namely, in the fusion zone the columnar grains were elongated along the thermal gradient direction, the heat affected zone (HAZ) consisted of the partially melted zone and the over-aged zone, and solidification cracks or liquation cracks which were quite normal in arc welding of aluminum alloys did not appeared, as shown in Figure 7. By comparison and contrast between Figure 7a,b, it was found that the width of the columnar zone in the arc zone was greater than that of in the laser zone because the cooling rate was faster due to the more efficient heat transfer in the arc zone. Moreover, the partially melted zone, which indicated the partial melting of the base aluminum alloys, in the arc zone (about 126 μm) was also wider that in the laser zone (about 107 μm) due to the high temperature residence time was longer in the arc zone [23]. The above differences were also observed in the plasma-CMT hybrid welded joint, as shown in Figure 7c,d. The widths of the partially melted zone both in the arc zone and laser zone of the laser-CMT hybrid welded joint was much narrower than that in the plasma-CMT hybrid welded joint, because of the higher heat input and lower welding speed during the plasma-CMT hybrid welding process. Moreover, the width ratio of the partially melted zone in the plasma zone and arc zone of the plasma-CMT hybrid welded joint (72.6%) was smaller than that in the laser-CMT hybrid welded joint (84.9%), which indicated the effects of plasma energy was a little weaker than that of laser energy.

### 3.2. Mechanical Properties

#### 3.2.1. Micro-hardness

**Figure 8:**
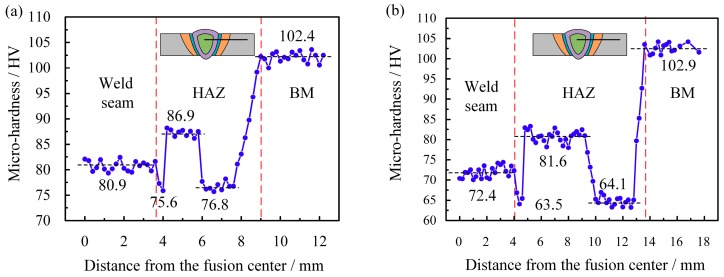
Micro-hardness profiles of the hybrid welded joints. (**a**) laser-CMT hybrid welded joint; (**b**) plasma-CMT hybrid welded joint.

Figure 8 shows the micro-hardness profiles across the laser-CMT and plasma-CMT hybrid welded joints. The indentations were carried out on the cross-section 2 mm below the weld top surface. It can be observed that, for both of the two hybrid welded joints, the micro-hardness values of the fusion zone and the heat affected zone decreased compared with that of the base material. Some existing research results show that the vaporization of alloying element and the grain size became larger, while the precipitates became less leaded to the reduction of the micro-hardness of the fusion zone and the heat affected zone, respectively [24,25,26]. As shown in Figure 8a,b, two distinct softening regions were identified in the heat affected zone of the two hybrid welded joints: one was in the partially melted zone because the partial melting of the grain boundaries resulted in the diffusion of strengthening alloying elements from solid phase to liquid phase with greater solubility [27]; the other was at a distance away from the fusion line (2.4–4.2 mm for laser-CMT hybrid welded joint and 5.8–8.8 mm for plasma-CMT hybrid welded joint), which, due to the obvious dissolution of β″ strengthening phases and the number of β″ phases, decreases substantially in this region [28]. By comparison, the micro-hardness values of each zone in the laser-CMT hybrid welded joint were lower than that in the plasma-CMT hybrid welded joint, and the width of the heat affected zone in the laser-CMT hybrid welded joint (5.4 mm) was much narrower than that of the plasma-CMT hybrid welded joint (9.8 mm), which were all because the heat input was higher during the plasma-CMT hybrid welding process.

#### 3.2.2. Tensile Strength

**Figure 9:**
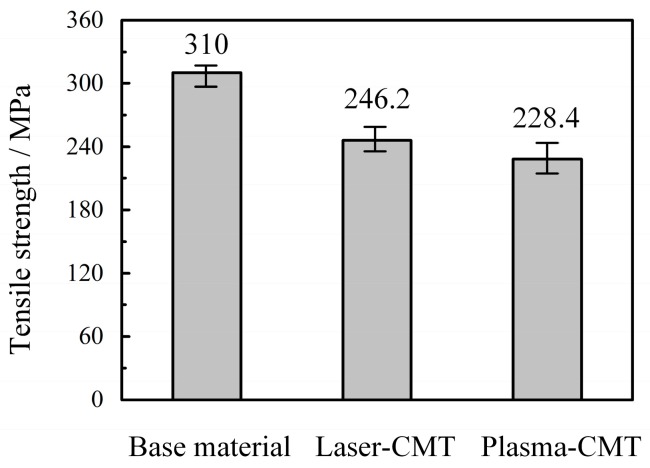
Tensile strength of base materials, laser-CMT, and plasma-CMT hybrid welded joints.

**Figure 10:**
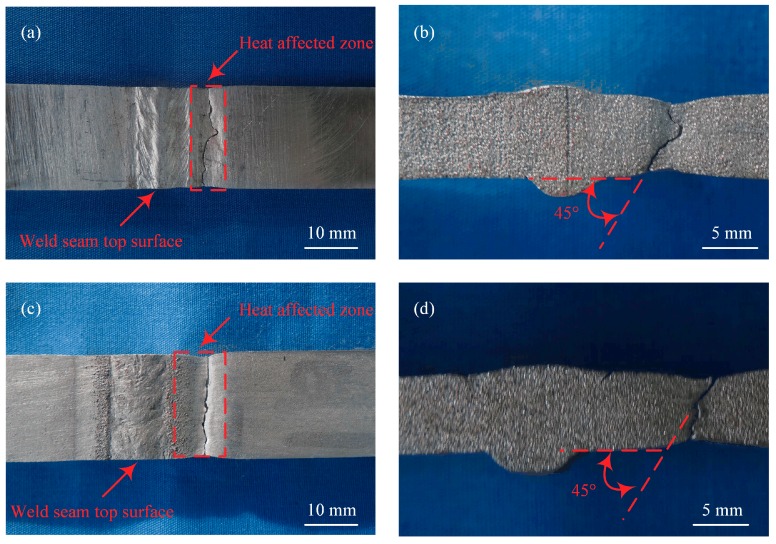
Fracture locations of the tensile test samples. (**a**) Top surface of laser-CMT hybrid welded joint; (**b**) cross-section of laser-CMT hybrid welded joint; (**c**) top surface of plasma-CMT hybrid welded joint; (**d**) cross-section of plasma-CMT hybrid welded joint.

The average tensile strength of the base materials, the laser-CMT hybrid welded joints, and the plasma-CMT hybrid welded joints as shown in Figure 9, three specimens were selected for each kind of tensile tests. The average tensile strength of the laser-CMT hybrid welded joints had reached up to 246.2 MPa and higher than that of the plasma-CMT hybrid welded joints (228.4 MPa), which were 79.4% and 73.7% of the base materials (310 MPa), respectively. The tensile strength were higher in case of laser-CMT hybrid welding than in the case of plasma-CMT hybrid welding was because the joint softened more seriously in the case of plasma-CMT welded joint. All the fractures of the tested laser-CMT and plasma-CMT hybrid welded samples occurred in the softening region of the heat affected zone, as shown in Figure 10a,c, which indicated this region was the weakest area of the hybrid welded joints. Meanwhile, the hybrid welded joints exhibited a ductile shear fracture with a shear angle of approximately 45° as shown in Figure 10b,d.

**Figure 11:**
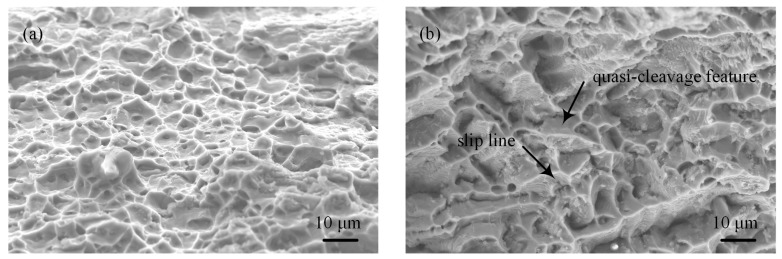
Fracture morphologies for the fracture surface of the hybrid welded joints. (**a**) Laser-CMT hybrid welded joint; (**b**) plasma-CMT hybrid welded joint.

Scanning electron microscope fractographs show that the fracture morphology exhibits different characteristics for the two kinds of hybrid welded joints. For the laser-CMT hybrid welded joint, the fracture surface shows ductile fracture appearance with lots of shallow and coarse dimples in the fractograph, as shown in Figure 11a. On the contrary, besides dimples, some slip lines and very few quasi-cleavage features were also observed in the fractograph of the plasma-CMT hybrid welded joint as shown in Figure 11b, which revealed that its fracture mode was a ductile-brittle mixed fracture and dominated as a ductile fracture.

## 4. Conclusions

(1) The overall quality of the laser-CMT and plasma-CMT welds was good. Compared to the plasma-CMT weld, the laser-CMT hybrid weld appeared “funnel shaped” and had a better surface forming quality. To obtain the same weld penetration, the laser-CMT hybrid welding process needed a lower heat input.

(2) Compared to plasma-CMT hybrid weld, the size of the equiaxed dendrites in the fusion center was smaller in the laser-CMT hybrid weld. The heat affected zone was consist of partially melted zone and over-aged zone in the two cases, and the width of the partially melted zone of the laser-CMT hybrid welded joint was narrower than that in the plasma-CMT hybrid welded joint; additionally, the width ratio of the partially melted zone in the plasma zone and arc zone of the plasma-CMT hybrid welded joint was smaller than that in the laser-CMT hybrid welded joint. 

(3) For both of the two hybrid welded joints, the micro-hardness values of the fusion zone and the heat affected zone decreased compared with that of the base material, and two distinct softening regions were identified in the heat affected zone. The micro-hardness values of each zone in the laser-CMT hybrid welded joint were lower than that in the plasma-CMT hybrid welded joint, and the width of the heat affected zone in the laser-CMT hybrid welded joint was much narrower than that of the plasma-CMT hybrid welded joint.

(4) The tensile strength of the laser-CMT hybrid welded joints was higher than that of the plasma-CMT hybrid welded joints, which could reach up to 79.4% and 73.7% of the base materials, respectively. All the fractures occurred in the softening region and exhibited a ductile shear fracture with a shear angle of approximately 45°. The laser-CMT hybrid welded joint showed obvious ductile fracture features, and the plasma-CMT hybrid welded joint presented ductile-brittle mixed fracture based on ductile fracture.

## Figures and Tables

**Table 1:** Chemical composition of the base material and filler wire (wt. %).

Materials	Si	Fe	Cu	Mn	Mg	Cr	Zn	Ti	Al
AA6082-T6	0.97	0.37	0.07	0.67	1.02	0.01	0.06	0.01	Bal.
ER5356	0.10	0.4	0.1	0.15	4.8	0.1	0.1	0.13	Bal.

**Table 2:** The optimized welding parameters adopted in the hybrid welding process.

Parameters	Laser-CMT Hybrid Welding	Plasma-CMT Hybrid Welding
Laser power/W	2000	
Plasma current/A		130
Plasma voltage/V		29
Plasma gas flow rate/l/min		7
CMT current/A	205	201
CMT Voltage/V	23	23
Welding speed/m/min	1.0	0.7
Wire feeding speed/m/min	12	9

**Table 3:** The geometric dimensions of each zone of the two hybrid welds.

Welding Method	*W*_A_/mm	*W*_L_*/W*_P_/mm	*D*_A_/mm	*D*_L_*/D*_P_/mm	*R*_F_/mm	*R*_B_/mm
Laser-CMT	7.48	3.46	2.97	2.99	0.76	1.61
Plasma-CMT	11.26	6.86	3.23	2.73	0.98	1.98
